# Moving beyond the neutral-Darwinian dichotomy to inform precision oncology

**DOI:** 10.1038/s41698-026-01606-8

**Published:** 2026-07-10

**Authors:** Amit Sud, Richard S. Houlston

**Affiliations:** 1 Centre for Immuno-Oncology, Nuffield Department of Medicine, University of Oxford, Oxford, UK; 2 Department of Medical Oncology, Dana-Farber Cancer Institute, Boston, MA USA; 3 Broad Institute of MIT and Harvard, Cambridge, MA USA; 4 Harvard Medical School, Boston, MA USA; 5 Division of Genetics and Epidemiology, The Institute of Cancer Research, London, UK

**Keywords:** Cancer, Computational biology and bioinformatics, Genetics, Oncology

## Abstract

Current methods for distinguishing neutral from selection-driven tumour evolution rely on assumptions frequently violated in clinical samples. We argue that tumour evolution is not a binary state, but a dynamic continuum shaped by genetic and non-genetic heterogeneity, spatial constraints, microenvironmental pressures and therapeutic bottlenecks. Refining evolutionary inference through integrative, longitudinal and spatially resolved analyses will be essential to align these models with therapeutic decision-making in precision oncology.

## Introduction

Cancer evolves dynamically as a consequence of elevated mutation rates, genomic instability and microenvironmental pressures, which together generate extensive genetic and phenotypic intratumour heterogeneity (ITH) that contributes to disease progression, metastasis and therapeutic resistance^1–4^. Evolutionary theory, therefore, provides a powerful conceptual framework for interpreting tumour diversity and understanding how cancer populations adapt over time.

**Fig. 1:**
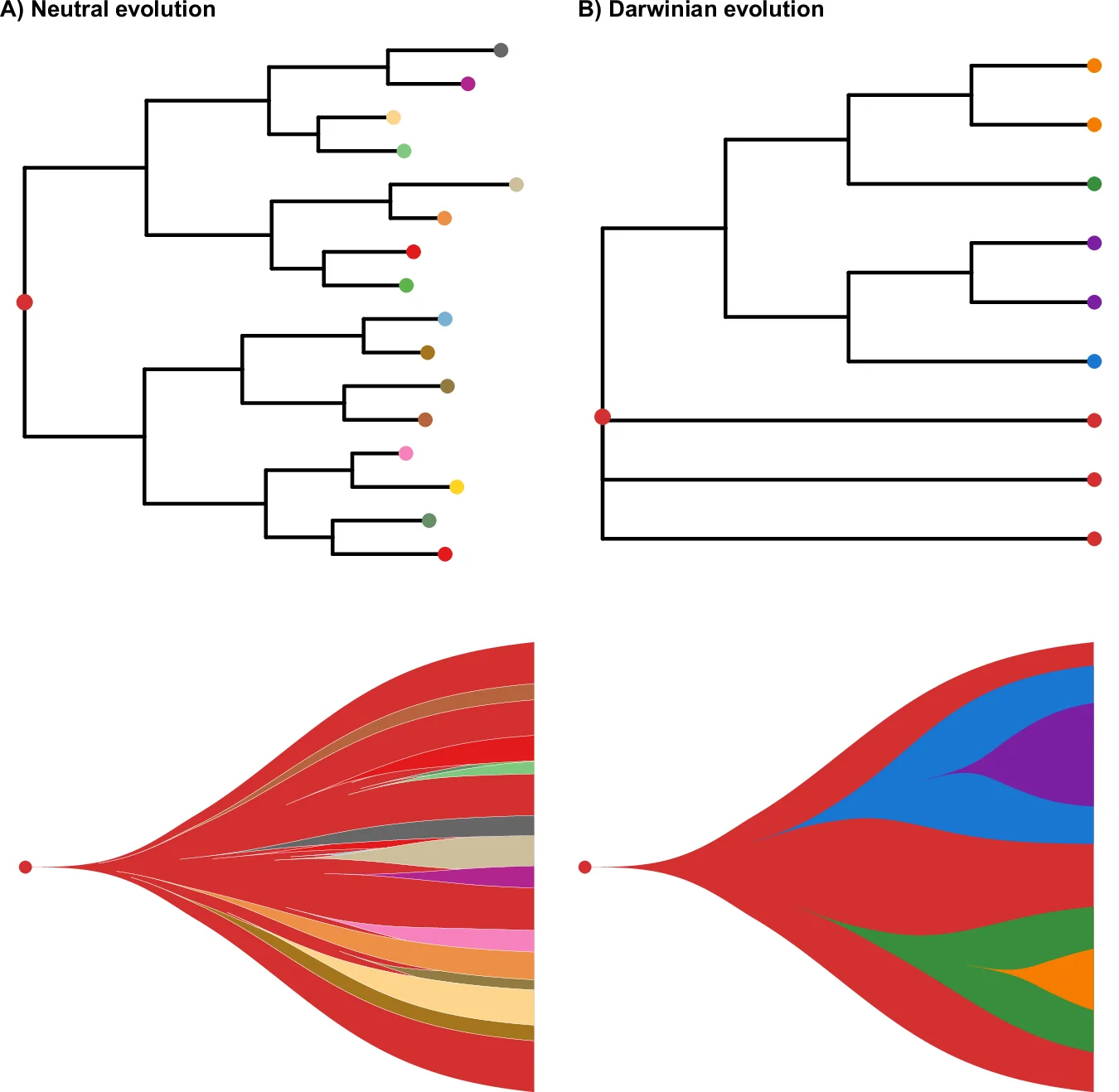
Schematic representation of neutral versus Darwinian tumour evolution. **A** Neutral evolution. Top: A balanced, rooted phylogenetic tree indicating equivalent fitness across simultaneously diversifying lineages. Bottom: The corresponding Muller plot illustrates the generation of extensive intratumour heterogeneity via genetic drift. The persistent ancestral population (red) demonstrates that early truncal driver mutations remain clonally shared across the tumour, while emergent passenger subclones (various colours) drift without competitive expansion. **B** Darwinian evolution. Top: An imbalanced phylogenetic tree characteristic of ongoing selection, reflecting sequential fitness advantages. Bottom: The Muller plot demonstrates successive selective sweeps. Fitter subclones (blue, green, purple, orange) undergo rapid, non-random expansion, competitively displacing the founding ancestral population (red) and pre-existing lineages over time.

The neutral theory of molecular evolution, first proposed by Motoo Kimura, in the context of population genetics, posits that most mutations are selectively neutral and accumulate through genetic drift rather than positive or negative selection^5^. Applied to cancer, this framework suggests that following the acquisition of early driver mutations, many subsequent somatic mutations behave as passengers, which do not confer a measurable fitness advantage. In such a scenario, clonal diversification arises largely through stochastic processes, allowing extensive genetic heterogeneity to accumulate without fundamentally altering tumour fitness^6,7^. The neutral evolution model therefore contrasts sharply with classical Darwinian frameworks, in which tumour evolution is directed by selection through clonal sweeps, branched evolution, or punctuated events such as chromothripsis (Fig. 1)^1,2,8^.

Understanding tumour evolutionary dynamics is not purely an academic exercise. If tumour growth approximates neutral expansion, most cancer cells would share a common set of early driver mutations, potentially simplifying therapeutic targeting. Conversely, tumours undergoing ongoing selection may harbour multiple competing subclones with distinct driver alterations, increasing the likelihood of therapeutic resistance. However, even tumours exhibiting patterns consistent with neutrality may contain rare resistant populations or phenotypically plastic cell states capable of driving relapse.

Evidence consistent with neutral dynamics includes observations of 1/*f* power-law distributions in variant allele frequencies^6^, *dN*/*dS* ratios approximating unity across coding mutations^9^, and star-like phylogenies reconstructed through multi-region sequencing^10^. Theoretical frameworks such as the “Big Bang” model have further proposed that many driver mutations arise early during tumour initiation, after which tumours expand rapidly with limited additional selection^7^. These data imply that in certain tumour types, especially those with low genomic complexity, subclonal diversification may proceed largely without selective pressure.

Although neutral evolution has been proposed as a pervasive feature of tumour development, this view remains contentious. The validity of such inferences has been challenged on the basis that apparent signatures of neutrality may arise from methodological limitations, spatially constrained growth, or unmodelled biological processes inherent to tumour evolution. Consequently, the true prevalence of neutral evolution in cancer remains uncertain, and distinguishing neutral from selection-driven evolution in clinical tumour samples remains a major methodological challenge. Here, we review the evidence supporting neutral evolution in cancer and evaluate methodologies used to detect it. We discuss how genomic complexity, spatial structure and non-genetic sources of heterogeneity complicate evolutionary inference from sequencing data. Finally, we consider how improved understanding of tumour evolutionary dynamics can reconcile these complexities into a unified evolutionary continuum which can inform precision oncology and guide the development of evolution-aware therapeutic strategies.

## Evidence for neutral evolution

### Power-law distribution of variant allele frequencies

**Fig. 2:**
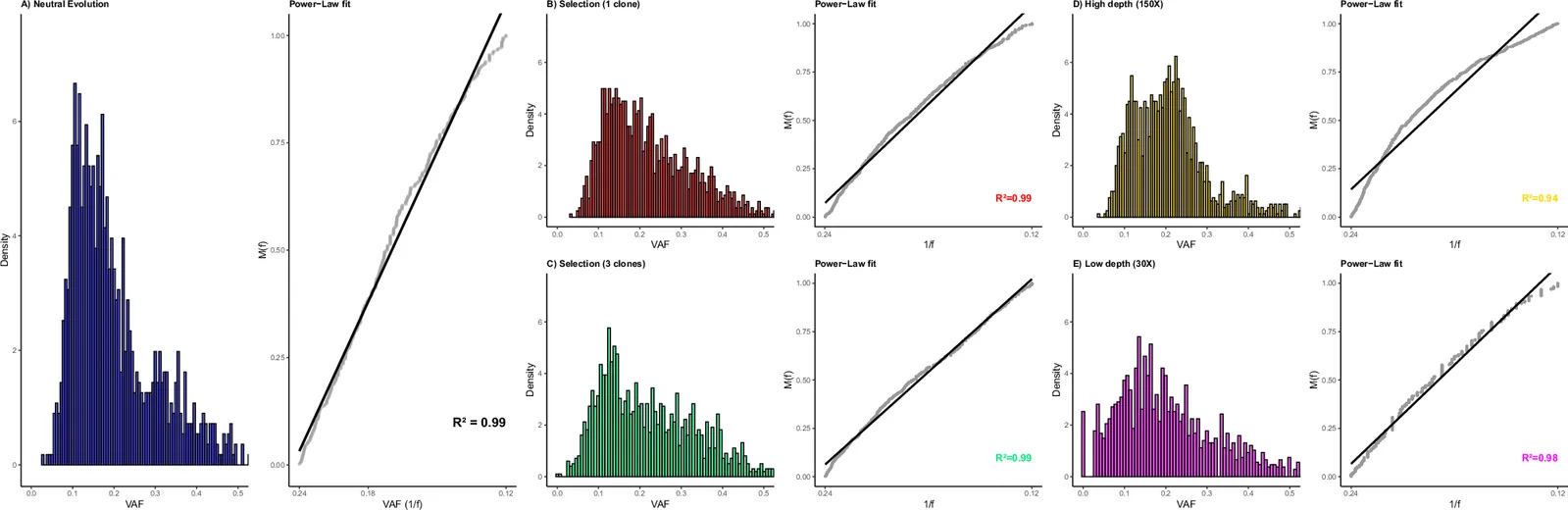
1/*f* Power-Law and biases. **A** Left: the VAF histogram displays a characteristic 1/*f* “long tail” of passenger mutations. Under constant birth/death rates and a lack of selective advantage, mutations accumulate stochastically as the population expands. Right: the cumulative frequency *M*(*f*) is plotted against the inverse frequency 1/*f*. A high coefficient of determination (*R*^2^ ≥ 0.98) indicates a near-perfect linear fit, correctly identifying the population as evolving under a near-neutral regime. **B** Despite a distinct selective sweep (VAF peak at ~0.25), the power-law test often yields an *R*^2^ ≥ 0.98. This illustrates that strong, recent selection can be mathematically indistinguishable from neutrality. **C** Despite a complex VAF distribution as a result of three co-existing subclones (VAFs 0.35, 0.25, 0.15), the cumulative power-law fit remains linear (*R*^2^ = 0.99), suggesting neutrality. **D** The VAF histogram at high sequencing depth resolves a distinct subclonal peak at VAF 0.22, indicating selection. The cumulative power-law fit deviates from linearity (*R*^2^ < 0.98), identifying the tumour as non-neutral. **E** Despite identical underlying biology, as **D** increased binomial sampling noise blurs the subclonal peak into the neutral tail. This smearing creates a smooth cumulative distribution that mimics a linear 1/*f* trajectory, resulting in a high goodness-of-fit (*R*^2^ ≥ 0.98) and a false positive classification of neutrality. Synthetic VAF distributions were generated via stochastic simulations in which neutral passenger mutations follow a uniform distribution of inverse frequencies (1/*f*) under Poisson-distributed sequencing depth. Subclonal populations were superimposed using beta-binomial sampling to model biological overdispersion and sampling noise at specific target frequencies. Goodness-of-fit was assessed via linear regression of the cumulative frequency *M*(*f*) against 1/*f* within the 0.12–0.24 frequency range.

One of the most widely cited lines of evidence supporting neutral evolution in cancer arises from analyses of variant allele frequency (VAF) distributions derived from tumour sequencing data^6^. In a neutrally evolving tumour undergoing exponential expansion, theoretical models predict that the cumulative number of mutations should scale inversely with their allele frequency, producing a characteristic 1/*f* power-law distribution in the site-frequency spectrum (Fig. 2A). In this statistical framework, newly arising mutations are assumed to confer no fitness advantage, such that all subclones expand at the same rate. Consequently, the allele frequency of a mutation is determined primarily by the number of cells present at the time the mutation occurs: mutations acquired earlier during tumour growth appear at higher frequencies, whereas those arising later remain at lower frequencies. Under these conditions, the cumulative number of mutations *M*(*f*) is predicted to follow the relationship *M*(*f*) ∝ 1/*f*. Strict adherence to this distribution, therefore, implies that ITH is generated largely through stochastic genetic drift rather than through selective expansion of distinct subclones.

Empirical support for this framework was reported by Williams et al., who analysed whole-exome and whole-genome sequencing data from 904 tumours across 14 cancer types in The Cancer Genome Atlas (TCGA)^6^. Approximately 32% of tumours were asserted to exhibit VAF distributions consistent with neutral dynamics. Neutrality was assessed by testing whether the cumulative mutation count followed the predicted relationship *M*(*f*) ∝ 1/*f* for mutations with VAFs between 0.12 and 0.25. Adherence to the model was evaluated using a goodness-of-fit threshold of *R*² ≥ 0.98 following attempted adjustments for tumour purity and ploidy. These findings were interpreted as supporting a model in which, after the early acquisition of driver mutations, subsequent mutations accumulate largely neutrally through genetic drift rather than through repeated selective sweeps. The validity of neutrality inferred from this modelling framework has been widely debated because several methodological assumptions may distort VAF distributions and lead to erroneous evolutionary inference (Table 1 and Fig. 2)^11–18^.

**Table 1:** Methodological challenges and mitigation strategies in the inference of neutral tumour evolutionary dynamics

Challenge	Impact on interpretation	Proposed solution
Assumption of a diploid genome	Misestimation of VAF distributions and false neutrality signals	Integrate accurate CNA calling and tumour purity estimates
Failure to account for CNAs, LOH and aneuploidy	Skews VAFs, leading to incorrect evolutionary inference	Use CNA-corrected VAFs and ploidy-aware models
Over-reliance on 1/*f* VAF fits (*R*² ≥ 0.98)	High false-positive rate for neutrality due to noise sensitivity	Employ statistical models with stricter thresholds and empirical validation
Limited spatial sampling (single-region analysis)	Misses spatial subclonal architecture and regional selection	Conduct multi-region sequencing to resolve spatial heterogeneity
Shallow sequencing depth	Insufficient detection of low-frequency subclones	Apply ultra-deep or single-cell sequencing for sensitive mutation detection
Ignoring non-genetic factors (epigenetic, microenvironmental)	Underestimates the role of plasticity and non-Darwinian dynamics	Integrate multi-omics (e.g., ATAC-seq, spatial transcriptomics)
Lack of longitudinal sampling	Cannot track temporal clonal shifts or therapy-induced selection	Perform serial biopsies or liquid biopsies over the treatment course

Confounding factors that can obscure true evolutionary signals. *VAF* variant allele frequency, *CNA* copy number alteration, *LOH* loss of heterozygosity, *ATAC-seq* assay for transposase-accessible chromatin with high-throughput sequencing.

### *dN*/*dS* analysis

Complementary evidence for neutral evolution in cancer has been sought using *dN*/*dS* analyses, which compare the rate of nonsynonymous (*dN*) to synonymous (dS) mutations to infer the action of natural selection^9^. As an established framework in molecular evolution, *dN*/*dS* analysis provides a quantitative and orthogonal approach for assessing selective pressures and can therefore complement VAF-based methods. Using this approach, Martincorena et al. analysed tumour genomes from TCGA and reported *dN*/*dS* ratios close to 1 across several tumour types within the coding regions of 192 well-established cancer genes^9^. These findings were interpreted as indicating an absence of strong positive or negative selection in these regions, consistent with the possibility that a substantial fraction of mutations accumulate neutrally during tumour evolution.

### Theoretical and computational modelling

Theoretical and computational models of tumour growth provide a conceptual framework for exploring the plausibility of neutral evolutionary dynamics. A prominent example is the “Big Bang” model proposed by Sottoriva et al., which suggests that many key driver mutations arise during the earliest phases of tumour initiation^7^. In this model, critical driver events are already present in the founding tumour cell population, after which the tumour undergoes rapid expansion. During this expansion phase, subsequent mutations accumulate largely neutrally as the tumour population increases in size. Under these conditions, stochastic mutation accumulation during tumour growth generates a characteristic 1/*f* site-frequency spectrum, consistent with VAF distributions observed in sequencing datasets.

Further theoretical support for this framework was provided by Tung et al., who used mathematical modelling and computational simulations involving large tumour cell populations (for example, on the order of 10⁹ cells)^19^. These simulations demonstrated that neutral growth dynamics could reproduce allele-frequency spectra closely resembling empirical VAF distributions derived from tumour sequencing data, providing a population-genetic rationale for neutral evolutionary patterns observed in some cancers.

## Methodological limitations of neutrality inference

### Limitations of 1/*f* VAF-based neutrality inference

A central limitation is the assumption of a diploid genome in the 1/*f* power-law model^12,14^. Under this assumption, clonal heterozygous mutations are expected to exhibit VAFs of approximately 0.5, whereas subclonal mutations cluster at lower VAFs (typically 0.12–0.25). In many cancers, however, genomic instability, including copy-number alterations (CNAs), loss of heterozygosity (LOH) and aneuploidy, distorts VAF distributions and compromises the distinction between clonal and subclonal variants. Copy-number alterations, which affect more than 30% of the genome in cancers such as ovarian and breast carcinomas^20^, substantially alter expected VAFs. For example, a heterozygous mutation in a triploid region would produce a VAF of approximately 0.33, whereas a homozygous mutation in a haploid region may reach a VAF of 1.0. Such distortions can lead to misclassification of clonal mutations as subclonal, inflating perceived subclonal diversity and improving apparent fits to a 1/*f* distribution even in tumours undergoing selection. While Williams et al. adjusted for tumour purity, they did not correct for locus-specific CNAs^6^. Subsequent analyses by Noorbakhsh et al. demonstrated that correcting for CNAs markedly reduces the number of tumours classified as neutral, particularly in malignancies with high CNA burdens^13^. Conversely, these same technical artefacts can lead to the over-calling of selection (false-positives). Just as CNAs can mask selection, they can also simulate it; for instance, a neutral passenger mutation occurring in a region of high-level focal amplification may present a localised VAF peak that mimics a high-fitness subclonal sweep. Loss of heterozygosity further complicates interpretation by altering allele representation. In cancers such as pancreatic and lung adenocarcinomas^20^, LOH can inflate retained allele frequencies or mask subclonal mutations entirely. Despite its prevalence, LOH is rarely incorporated into 1/*f* analyses, increasing the risk of false-positive neutrality classifications, particularly in tumours with extensive LOH burdens such as head and neck squamous cell carcinoma. Aneuploidy introduces additional complexity because changes in chromosome or chromosome-arm number alter baseline ploidy. For example, in a tetraploid tumour, a clonal heterozygous mutation would present a VAF of approximately 0.25 and could therefore be misclassified as subclonal. Moreover, bulk sequencing averages allele frequencies across heterogeneous cell populations, obscuring regional ploidy variation. Tools such as ABSOLUTE can estimate average tumour ploidy but often lack sufficient resolution to capture local heterogeneity^21^, particularly in tumours with extensive genomic instability such as high-grade serous ovarian cancer^20^. Collectively, these genomic complexities confound the essential task of distinguishing clonal from subclonal mutations, a prerequisite for accurate 1/*f*-based neutrality analysis.

The statistical criterion used to classify tumours as neutral has also been widely criticised. Williams et al. defined neutrality using a fixed goodness-of-fit threshold of *R*² ≥ 0.98^6^. However, this threshold is relatively permissive and does not uniquely distinguish neutral from selection-driven evolutionary dynamics^11,13^. Tarabichi et al. showed that tumours containing two to four subclones evolving under moderate selective pressures (approximately 1–5% fitness advantage) can produce VAF distributions closely resembling 1/*f* power laws, yielding *R*² values between 0.90 and 0.99 (Fig. 2B, C)^14^. These findings indicate that a high *R*² value alone is insufficient evidence for neutrality, as similar statistical fits may arise in tumours undergoing branched evolution or weak positive selection. Similarly, Noorbakhsh et al. showed that CNAs broaden the subclonal tail of the VAF distribution, artificially improving apparent 1/*f* fits and inflating *R*² values^13^.

Sequencing depth introduces an additional source of bias. The relatively low coverage typical of datasets such as TCGA (30–60×) introduces considerable stochastic noise that smooths VAF distributions and can produce spuriously high *R*² values (Fig. 2D, E). High-depth sequencing (>100×) is required to reliably detect rare subclonal variants and deviations from neutral expectations^11^. Limited sequencing depth also reduces sensitivity to weak or transient selection. As noted by Bozic et al., mutations with small fitness effects may evade detection by 1/*f* models, and Tarabichi et al. confirmed that weak selection (<5%) can generate power-law-like patterns, particularly when analyses rely on single-time-point data^14,22^.

Beyond these statistical considerations, the deterministic growth model underlying the 1/*f* framework relies on strong biological assumptions that rarely hold in real tumours. The model assumes synchronous cell division, constant cell death rates and stable mutation and division rates, whereas tumour growth is highly stochastic, generations overlap and mutation rates may fluctuate because of genomic instability. Reliance on single-time-point bulk sequencing provides only a static snapshot and cannot capture temporal shifts, so apparent pre-treatment neutrality may give way to therapy-driven selective sweeps, and longitudinal sampling is required to track clonal trajectories^23^. A focus on genomic mutations overlooks non-genetic contributors to ITH, including epigenetic and microenvironmental phenotypes that confer fitness independently of mutational status, as in chronic lymphocytic leukaemia (CLL)^24^. Finally, spatial structure further complicates evolutionary inference. Multi-region sequencing studies have demonstrated extensive regional heterogeneity within tumours, revealing branched evolutionary patterns that cannot be detected from single-biopsy samples^23,25^. Modelling studies further indicate that tumours growing through local expansion and limited cell dispersal can generate allele-frequency distributions resembling neutrality even when selective differences exist between clones^26^.

Taken together, these limitations show that adherence to a 1/*f* distribution with a high R² value is compatible with neutral evolution but does not establish it, since similar patterns arise under several non-neutral scenarios. Apparent power-law behaviour should therefore be interpreted cautiously, and more integrative approaches are required to characterise tumour evolution accurately.

### Limitations of *dN*/*dS-*based neutrality inference

Interpretation of *dN*/*dS* in cancer genomics differs from classical evolutionary contexts and is subject to several methodological constraints. In many analyses, mutations are aggregated across genes or tumour cohorts to increase statistical power^9,27^. Although this improves the ability to detect broad patterns, it can obscure gene-specific signals of selection and dilute evidence of positive selection occurring within small subsets of genes or individual tumour subclones. Consequently, signals of selection that are spatially restricted or confined to specific evolutionary lineages may be masked when mutations are analysed collectively.

Additional caveats further constrain the interpretation of *dN*/*dS* estimates in tumours. Many analyses are restricted to known cancer genes, potentially overlooking selection acting in non-coding regions, structural variants, or previously unrecognised oncogenic loci. Mutational-process heterogeneity across tumour types and genomic contexts may also bias estimates by altering the underlying mutation spectrum. Furthermore, copy-number alterations can lead to misclassification of mutations as clonal or subclonal, thereby influencing inferred selection signals and distorting *dN*/*dS* estimates.

Statistical power represents another important limitation. Tumours with relatively low mutational burdens, such as early-stage or indolent cancers, may contain too few mutations to reliably detect weak or context-dependent selection. Consequently, observed *dN*/*dS* ratios close to unity do not necessarily provide definitive evidence for neutrality and may instead reflect methodological constraints or limited statistical sensitivity.

### Limitations of theoretical and computational models

Despite their mathematical rigour, these models inevitably simplify the biological complexity of tumour evolution. Many analytical frameworks rely on assumptions that are rarely fully satisfied in real tumours. For example, models often exclude key variables such as microenvironmental selection pressures, including hypoxia and immune evasion, non-genetic mechanisms of therapy resistance and spatial constraints on tumour growth. In addition, theoretical formulations frequently rely on an “infinite sites” assumption, in which each mutation arises only once. In very large tumours, this assumption is likely to be violated, leading to recurrent mutations that confound model predictions^28^. The limited incorporation of CNAs further restricts the applicability of these models, particularly in genomically unstable tumours.

Consequently, although theoretical and computational models demonstrate that neutral dynamics are biologically plausible and can reproduce patterns observed in sequencing data, their interpretation remains subject to considerable debate. Apparent signatures of neutrality may arise from methodological artefacts or incomplete modelling of tumour biology. These frameworks may therefore approximate evolutionary trajectories in certain contexts, such as early-stage tumours or some haematological malignancies; their relevance to advanced, spatially heterogeneous, and genomically complex tumours remains uncertain.

## Beyond neutral versus selection: a continuum of evolutionary trajectories

**Fig. 3:**
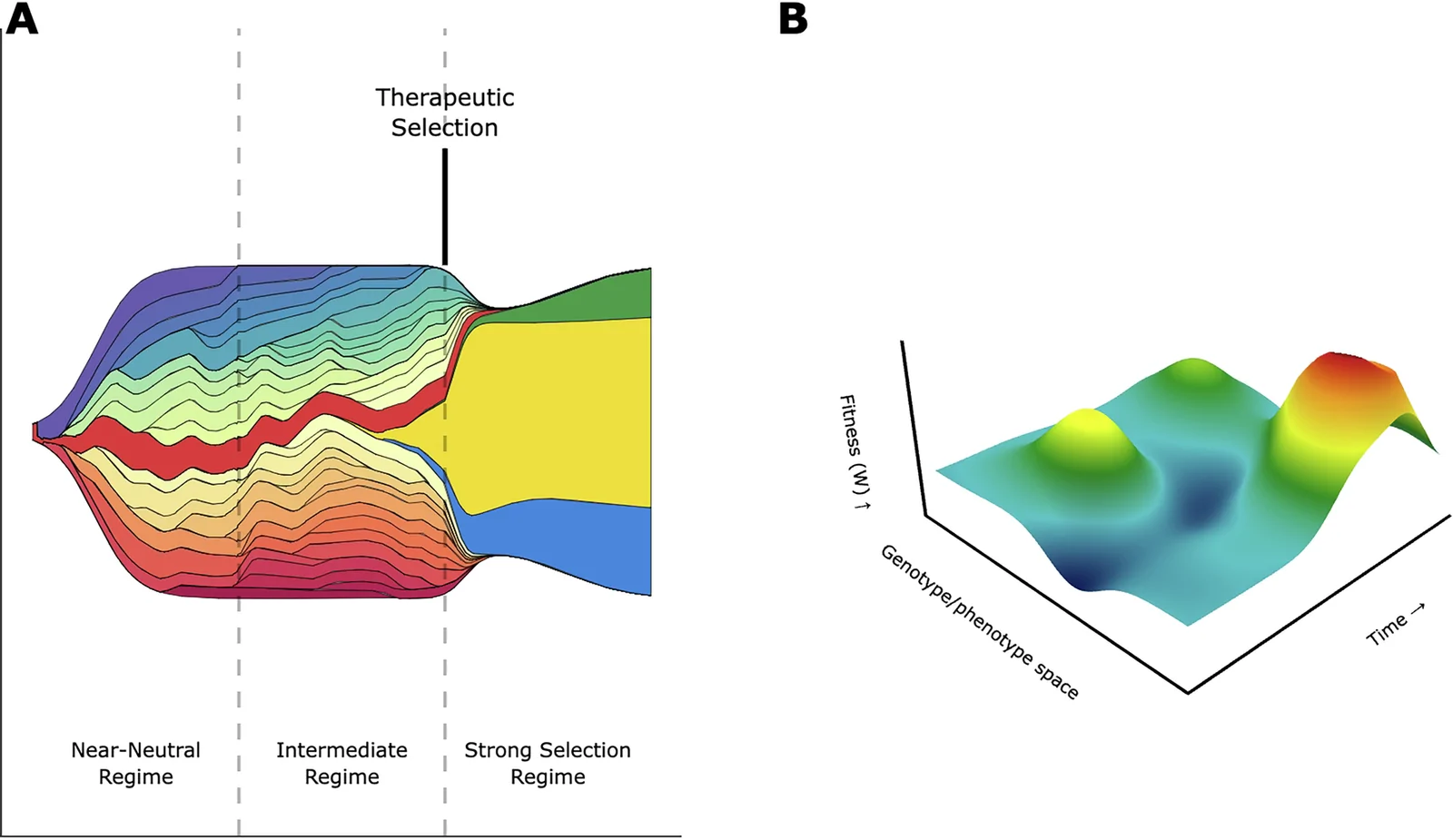
The genotype–phenotype-temporal dynamics of the evolutionary continuum. **A** Tumour evolution is represented here not as a strict neutral–Darwinian dichotomy, but as a dynamic continuum. Near-neutral regime (left): Following the acquisition of early founder mutations (red), subsequent mutations accumulate primarily via genetic drift. During this phase, subclones expand at nearly equivalent rates, resulting in high intratumour heterogeneity driven by stochastic processes during initial population expansion. Intermediate regime (centre): Characterised by weak or context-dependent selection. Modest fitness differences exist between clones, but their effects are often attenuated by spatial constraints and microenvironmental heterogeneity. In bulk sequencing, these subtle selective shifts are frequently masked by the background of neutral diversification. Strong selection regime (right): Advantageous mutations confer substantial fitness benefits, leading to rapid clonal expansion. This phase, often triggered or intensified by therapeutic selection (indicated by the arrow), results in a dramatic reshaping of the architecture, where dominant resistant subclones (gold, blue and green) execute selective sweeps that compress pre-existing diversity. **B** The incorporation of phenotypic dimensionality, encompassing spatial constraints, microenvironmental niches and epigenetic states, adds a complex topography to the fitness landscape. Axis Definitions: The horizontal axis represents the genotype-phenotype space, reflecting the combined influence of genetic alterations and functional traits (e.g., metabolic adaptation or spatial positioning). The depth axis represents temporal progression, showing how the landscape evolves over the life of the tumour. The vertical axis represents cellular fitness (W), where height corresponds to the reproductive success and survival advantage of a given lineage. Topographic features: The Neutral Plateau (Teal/Grey): A broad, relatively flat region where mutational “movement” does not result in significant vertical (fitness) gains, representing the near-neutral regime where drift dominates. Rolling Hills & Ridges (Green/Blue): These represent the emergence of selection. Clones begin to climb these peaks as they acquire mutations in a specific context that provide context-dependent advantages. Chopped Peaks (Red): The highest points of the landscape, representing reaching a fitness optimum. The “chopped” or flattened summits visually signify fitness canalisation, where a clone has reached a maximum level of adaptation within its niche, and further mutations provide no additional selective benefit. The Therapeutic Trench (Deep Blue): The deep valley represents the intense selective pressure of treatment, which effectively reduces the fitness of the majority of the population, leaving only those lineages on the resistant peaks viable.

A fundamental insight emerging from these limitations is that no single metric can reliably classify tumours as neutral or Darwinian when temporal and spatial heterogeneity in evolutionary mode are pervasive: a tumour may evolve near-neutrally in one region while selection operates in another, or may shift between regimes over time and under therapeutic pressure. This continuum of evolutionary trajectories, rather than a fixed binary state, thus provides the strongest conceptual argument against the dichotomy critiqued above. Although the neutral-versus-selection distinction has been conceptually useful, increasing theoretical and empirical evidence suggests it is overly simplistic, and recent work has shifted the field toward spatially structured, temporally dynamic and multi-factorial models of tumour evolution (Fig. 3).

Spatial constraints profoundly influence detectable patterns of selection and drift. Tumour growth geometry, local dispersal and microenvironmental barriers can generate allele-frequency distributions resembling neutrality even in the presence of underlying fitness differences. Spatially explicit simulations demonstrate that boundary-driven versus exponential expansion can produce neutral-like VAF distributions or apparent selection signals even under weak fitness differences^29^. Indeed, distinct spatially mediated evolutionary modes such as rapid clonal expansion, progressive diversification, branching and neutral-like, illustrate how cell mixing and physical barriers govern clonal architecture independently of genetics^30^. Conversely, boundary-driven growth or spatial bottlenecks can amplify modest selective advantages, producing apparent clonal dominance. These observations highlight that spatial structure can both mask and exaggerate signals of selection, complicating evolutionary inference from bulk sequencing data.

Ecological interactions within the tumour microenvironment further shape these trajectories. Selective pressures, including hypoxia, immune surveillance, stromal interactions and metabolic constraints, vary across tumour regions and may favour distinct phenotypic states in different locations. For instance, spatial phylogeography in hepatocellular carcinoma reveals block-shaped heterogeneity with large genotypic and phenotypic differences across tumour sectors, consistent with selection-driven compartmentalisation rather than uniform neutral drift^31^. This compartmentalisation is further illuminated by pan-cancer spatial transcriptomics, which demonstrates that tumours are organised into structurally distinct regions^32^. More recently, high-resolution platforms integrating single-nucleus transcriptomics with targeted genotyping and T cell receptor sequencing have mapped these interactions at the clonal level. Such approaches reveal a spatially organised anti-tumour immune response, where clonally expanded, neoantigen-specific T cells colocalise with tumour subclones expressing their cognate neoantigens. This physical proximity directly dictates T cell avidity, functional state and local immune niche composition, creating immune ‘hot’ and ‘cold’ neighbourhoods that exert highly localised selective pressures on individual clones^33^. Consequently, fitness advantages conferred by specific mutations may be weak, transient, or context-dependent. Subclones effectively neutral in one microenvironment may gain a selective advantage following metabolic stress, immune evasion, or therapeutic intervention. This context-dependent fitness landscape implies that tumours may cycle between near-neutral diversification and periods of positive selection.

Non-genetic heterogeneity adds an additional layer of complexity. Epigenetic states, transcriptional plasticity and phenotypic switching can alter cellular fitness independently of underlying mutational status. In colorectal cancer, for example, intratumour genetic ancestry infrequently drives gene expression traits or subclonal progression, with plasticity enabling rapid adaptation independent of somatic mutations^34^. This non-mutational fitness can be shaped by historical environmental exposures. Recent high-resolution lineage tracking reveals that colonic stem cells retain a clonally heritable ‘epigenetic memory’ of chronic inflammation, characterised by durable changes in chromatin accessibility and AP-1 transcription factor activity. This memory is propagated cell-intrinsically and primes specific clones for accelerated tumour growth upon subsequent oncogenic mutation, demonstrating how persistent epigenetic states can dictate subclonal fitness^35^. In such cases, selective differences arise through heritable but non-mutational mechanisms, meaning that analyses based solely on VAFs capture only the genetic substrate of tumour heterogeneity. As a result, VAF-based approaches may misclassify evolutionary dynamics when significant fitness variation arises from epigenetic or environmentally induced phenotypes.

Temporal dynamics further underscore the continuum of evolutionary regimes. Early tumour growth often involves strong selection for initiating driver mutations, followed by periods of relative evolutionary stasis, during which additional mutations accumulate with limited fitness effect. Later stages, particularly under therapeutic pressure, can impose intense exogenous selection that dramatically reshapes clonal architecture. Importantly, evidence of selection following therapy does not necessarily indicate that tumour evolution before treatment was dominated by selection. Longitudinal sampling, including serial biopsies or circulating tumour DNA analyses, is therefore critical to distinguish endogenous evolutionary patterns from the profound shifts induced by clinical intervention. These therapeutic pressures represent a definitive selective bottleneck, fundamentally altering clonal trajectories. This is illustrated by the emergence of treatment-specific driver mutations, such as the *EGFR* T790M mutation in non-small cell lung cancer and *ESR1* alterations in breast cancer, which are often undetectable or at low VAF in treatment-naïve disease but rapidly sweep through the population under therapeutic pressure^36–38^. Longitudinal sequencing across various malignancies has further characterised these selective bottlenecks. In CLL, chemotherapy results in large clonal shifts while facilitating the competitive release of rare, resistant subclones (*e.g*., those harbouring *TP53* mutations) that subsequently dominate at relapse^39,40^. Similar dynamics are seen in acute myeloid leukaemia (AML), where the therapeutic pressure of induction therapy selects minor subclones rather than the founding clone^41^. Furthermore, large-scale studies such as TRACERx have demonstrated that metastatic seeding and systemic therapy can lead to clonal sweeps where a single evolutionary branch dominates distant sites despite high primary intratumour heterogeneity^42^. As such, pre-treatment neutrality does not reliably predict post-treatment behaviour; selective bottlenecks introduced by cytotoxic or targeted therapies can convert neutral-like patterns into resistant states, sometimes reducing detectable selection signals while promoting poor clinical outcomes. High-resolution approaches such as single-cell multi-omics, spatial transcriptomics and longitudinal liquid biopsies promise to capture these dynamics, although technical and practical constraints on scale, cost and integration persist. Bayesian and likelihood-based methods applied to bulk data provide complementary robustness, enabling probabilistic evaluation of neutrality versus selection without ultra-deep sequencing.

Taken together, emergent observations indicate that neutrality and selection should not be viewed as mutually exclusive states, but rather as context-dependent approximations that describe different phases or aspects of tumour evolution. The critical challenge is not simply to classify tumours as neutral or Darwinian, but to determine when neutral models sufficiently describe clonal dynamics and when deviations from neutrality meaningfully influence tumour behaviour, therapeutic response and clinical outcomes.

## Clinical implications of evolutionary inference

Determining whether tumour growth approximates neutral dynamics or is shaped by ongoing Darwinian selection has potential implications for therapeutic strategy and clinical trial design. However, the clinical relevance of evolutionary classification depends critically on whether it alters decision-making rather than serving as a purely descriptive label. Importantly, methodological limitations, including reliance on permissive statistical thresholds, uncorrected CNAs or low-depth sequencing data, may lead to selection-driven tumours being misclassified as neutral. These analytical artefacts raise the risk of therapeutic misdirection.

From a theoretical standpoint, tumours approximating neutral expansion should exhibit high subclonal diversity composed largely of passenger mutations, while most tumour cells share truncal driver alterations. In such settings, targeting early clonal drivers may achieve broad tumour coverage as the probability of pre-existing strongly selected resistant subclones is theoretically lower. This framework has been invoked to justify therapeutic de-escalation strategies or reliance on single-agent targeted therapy in certain contexts. If robustly validated, this approach could enable treatment stratification wherein therapy focuses on truncal drivers while avoiding unnecessary treatment intensity. For example, neutral tumours might respond durably to targeted agents, such as tyrosine kinase inhibitors in chronic myeloid leukaemia or immune checkpoint inhibitors in microsatellite-instable cancers^43,44^, and some patients might benefit from maintenance approaches rather than intensive chemotherapy.

Nevertheless, neutrality does not preclude therapeutic resistance. Even in tumours exhibiting VAF distributions consistent with neutral drift, rare subclones with modest fitness advantages may exist below detection thresholds. Furthermore, phenotypic plasticity and epigenetic reprogramming can generate resistance without the acquisition of new mutations. Consequently, classification of a tumour as “neutral” should not be interpreted as evidence that resistance is unlikely or that combination therapy is unnecessary. Conversely, tumours demonstrating clear evidence of ongoing positive selection and branched evolution harbour multiple subclonal driver mutations defining distinct evolutionary lineages. In this setting, monotherapy targeting a single pathway risks competitive release, whereby elimination of therapy-sensitive clones accelerates expansion of resistant populations. Evolution-aware strategies may therefore be more appropriate. These include upfront combination therapies designed to extinguish subclonal diversity, or adaptive dosing strategies intended to maintain sensitive competitors and suppress resistant clones, a concept sometimes described as evolutionary “shepherding”^45^. The clinical consequences of evolutionary misclassification are therefore asymmetric and context-dependent. Falsely assuming neutrality in a tumour harbouring undetected resistant subclones may lead to undertreatment and rapid relapse, whereas incorrectly inferring strong selection may prompt overtreatment, exposing patients to unnecessary toxicity without clear benefit. The relative risk of these errors varies by tumour type, disease stage, therapeutic modality and treatment intent, whether curative or palliative.

Despite the theoretical appeal of evolution-aware therapeutic strategies, their implementation in routine oncology remains challenging^46^. Cancer progression is inherently stochastic, non-genetic mechanisms contribute substantially to resistance, and only a limited number of effective therapeutic options may be available for many malignancies. Practical constraints on tumour sampling mean that comprehensive, real-time monitoring of the entire tumour burden is rarely feasible. Without improved biomarkers and more sensitive methods for detecting clinically relevant subclones, predicting the precise evolutionary trajectory of an individual patient remains difficult. Moreover, given that therapeutic intervention fundamentally reshapes the tumour fitness landscape^25,47^, disentangling endogenous tumour evolution from therapy-imposed selection requires longitudinal sampling, including serial biopsies or circulating tumour DNA analysis. Such strategies are essential for understanding how clonal architecture evolves over time and for identifying emerging resistant populations before clinical relapse.

Ultimately, evolutionary classification will only influence clinical practice if it improves the prediction of resistance, informs the choice between monotherapy and combination therapy, guides treatment intensity, or determines the frequency of disease monitoring. Integrating genomic, spatial and longitudinal data will therefore be necessary before evolutionary models can be deployed reliably in routine cancer care.

## Recommendations for future research

Future work on neutral evolution in cancer should focus on developing integrative and biologically realistic approaches that better reflect the spatial, ecological and temporal complexity of tumour growth. As established, the intrinsic constraints of bulk sequencing often mask asexual dynamics, which confounds our ability to resolve the underlying fitness landscape. In cancer populations, genetic linkage leads to extensive Hill–Robertson interference, where beneficial drivers and deleterious passengers co-segregate within expanding clones^48,49^. Under these conditions, mildly deleterious mutations can accumulate through mechanisms resembling Muller’s ratchet, especially when purifying selection is inefficient. Because VAF-based metrics inherently condense this complex fitness information into simple frequency distributions, they are often unable to discriminate between genuine neutrality, positive selection with hitchhiking, or ratchet-like mutational degradation.

This challenge of identifiability underscores the necessity for analytical frameworks that move beyond the 1/*f* null model. Bayesian, likelihood-based, or mechanistic, future models must more explicitly incorporate spatial structure, growth geometry and the genomic landscape to resolve the respective roles of drift, selection and mutational load^30,50^. A major challenge is the detection of weak, transient, or context-dependent selection. Many bulk-sequencing methods lack the power to resolve fitness differences below a modest threshold, meaning that failure to detect selection does not imply its absence. Improving this sensitivity, while simultaneously determining when weak selection is clinically meaningful, will require models that jointly infer selection coefficients, clonal mixing and spatial dynamics. To capture the full spectrum of heritable fitness, future studies must integrate genomic profiling with epigenomic and spatial transcriptomics, identifying fitness variants that escape VAF-based detection entirely.

Empirical progress will depend heavily on the broader application of multi-region, single-cell and spatial transcriptomic technologies. Systematic sampling across tumour types, stages and microenvironmental contexts will be essential for determining where along the neutrality–selection continuum human cancers typically lie. Longitudinal studies represent a further priority, as static snapshots cannot capture temporal shifts between neutral and selective phases or distinguish pre-treatment evolutionary dynamics from therapy-imposed selection. Serial sampling using tissue biopsies or circulating tumour DNA will be critical for understanding how fitness landscapes change during treatment and for linking evolutionary trajectories to therapeutic response^37,51^.

A key objective moving forward is to define when and how evolutionary classification should inform clinical decision-making. Neutrality becomes clinically relevant only when it meaningfully influences choices such as the use of monotherapy versus upfront combination therapy, the identification of truncal therapeutic targets, or the intensity of surveillance required to detect emerging resistance. Prospective clinical studies incorporating evolutionary biomarkers will be needed to determine whether classifying tumours along a neutrality–selection continuum improves prediction of treatment response, risk of relapse, or progression under targeted or adaptive therapies.

Finally, computational inference should be paired with experimental validation. Organoid models, lineage-tracing systems and CRISPR-based perturbation platforms offer opportunities to test theoretical predictions under controlled spatial and ecological conditions, enabling a more rigorous evaluation of when neutral models provide an adequate approximation and when selective forces dominate^52,53^. Together, these efforts will help establish a more mechanistically grounded and clinically actionable framework for interpreting tumour evolution, ensuring that neutrality is applied neither prematurely nor dismissed without sufficient evidence.

## Conclusion

Neutral evolution provides a useful conceptual and statistical null model for interpreting ITH, but its prevalence and biological significance in human cancer remain uncertain. Patterns that have been interpreted as consistent with neutral dynamics have been reported in some tumour contexts. However, these signals must be interpreted cautiously. Assumptions such as diploid genomes, uniform exponential growth, permissive goodness-of-fit thresholds and incomplete correction for CNAs can inflate estimates of neutrality. In addition, spatial growth constraints, genomic complexity and sampling limitations may generate allele-frequency patterns that resemble neutral drift even when selection is operating. Equally, failure to detect selection does not imply its absence. Weak fitness differences, limited sequencing depth and non-genetic sources of phenotypic variation can obscure selective processes. A growing body of work, therefore, suggests that tumour evolution rarely conforms strictly to either neutral or purely Darwinian models. Instead, evolutionary dynamics likely exist along a continuum shaped by spatial growth patterns, ecological interactions, genomic instability, microenvironmental pressures and therapeutic perturbations. Under this framework, tumours may undergo periods of near-neutral diversification interspersed with phases of stronger positive selection, with distinct evolutionary regimes potentially occurring across tumour regions or over time. The clinical implications of evolutionary classification will depend on whether evolutionary inference meaningfully improves the prediction of therapeutic response or resistance. Overinterpreting apparent neutrality risks overlooking clinically relevant subclonal drivers or non-genetic resistance mechanisms, whereas overcalling selection may prompt unnecessarily aggressive treatment strategies. Consequently, more robust methods for evolutionary inference are required before such frameworks can reliably inform clinical decision-making. Advances in spatial genomics, high-depth sequencing, longitudinal sampling and integrative multiomic analyses will be essential to clarify when neutrality provides a useful approximation of tumour growth. Ultimately, improving the accuracy and interpretability of evolutionary models is essential if evolutionary principles are to be translated into clinically actionable strategies to advance precision oncology^54^.

## Data Availability

No datasets were generated or analysed during the current study.
